# Supplementary figures and images for: Host prion protein expression levels impact prion tropism for the spleen
Source: PLoS Pathog. 2020 Jul 23;16(7):e1008283. doi: 10.1371/journal.ppat.1008283 (PMC7402522; doi:10.1371/journal.ppat.1008283)

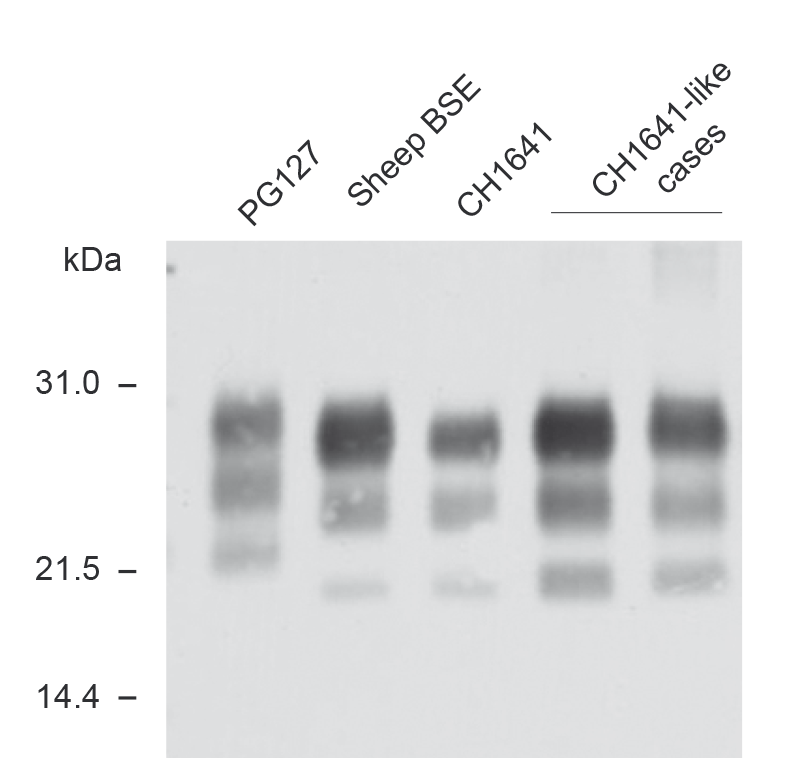

Supplement: S1 Fig — Electrophoretic pattern of PrPres in the brain from CH1641-like cases, compared with CH1641 isolate [36], PG127 isolate [71] and sheep experimentally inoculated with BSE prions [76]. (TIF) [file ppat.1008283.s001.tif]

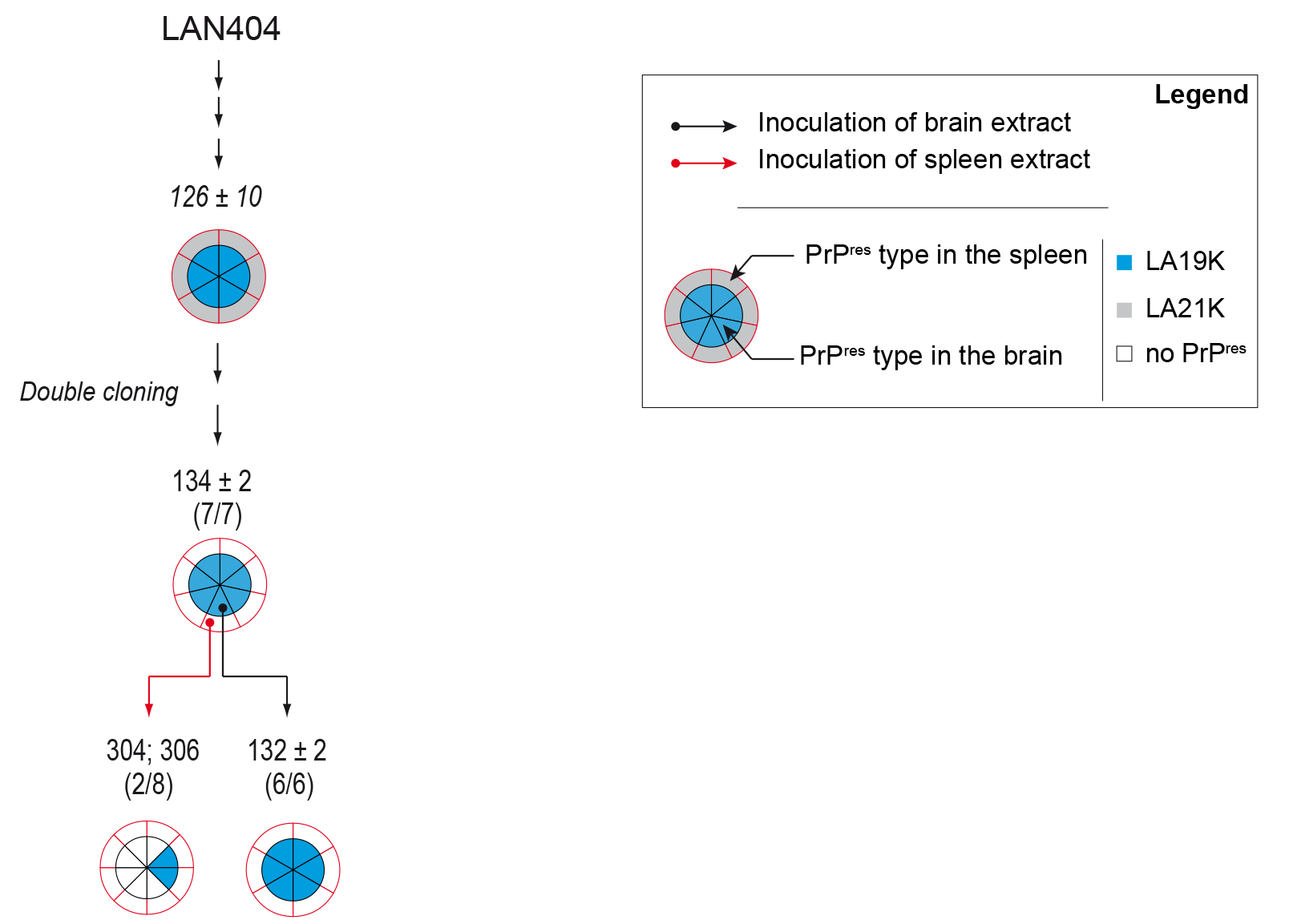

Supplement: S2 Fig — Cloned LA19K prions were obtained by serial passage of one LAN isolate (LAN404) and bicloning by limiting dilution in tg338 mice [29]. Brain or spleen extracts from tg338 mice infected with cloned 19K prions were inoculated (IC route) to reporter tg338 mice. Transmission with brain or spleen extracts are indicated with black and red lines, respectively. The number of affected/inoculated mice (mice with TSE and positive for brain PrPres by immunoblot) and the mean survival times in days ± SEM are indicated for each inoculated group. Segmented, doubled circles are used to indicate the proportion of mice with 19K PrPres signature (blue), 21K PrPres signature (grey) or absence of PrPres (white), either in the brain (inside of the circle, black lines) or in the spleen (outside of the circle, red lines). The data shown are representative of 5 independent transmission experiments with different mice infected with cloned LA19K prions. Data in italic are from [29]. (TIF) [file ppat.1008283.s002.tif]

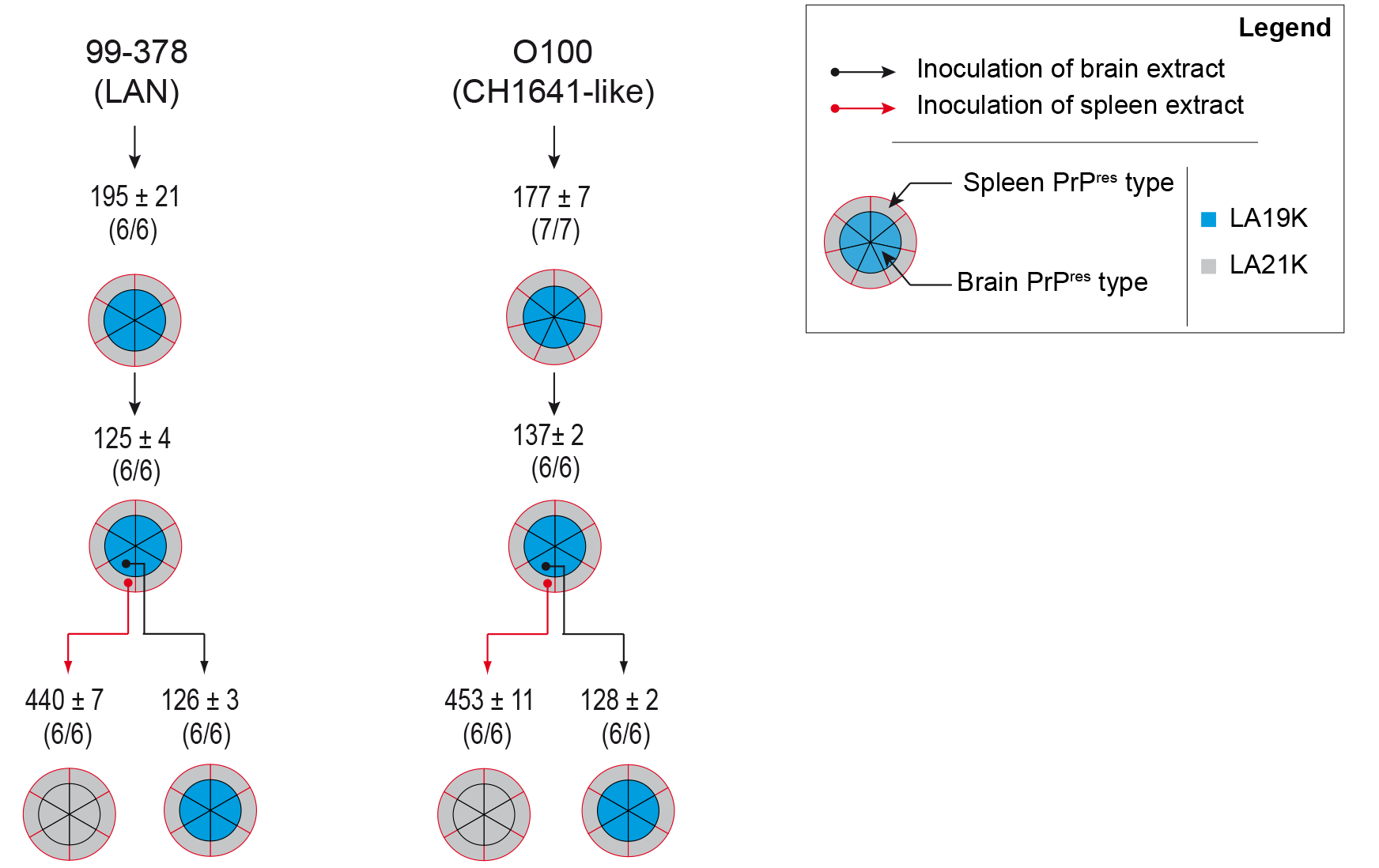

Supplement: S3 Fig — Transmissions by IC route of brain or spleen extracts from tg338 mice infected with the LAN isolate (99–378 isolate, 2nd passage) or the CH1641-like isolate (O100 isolate, 2nd passage) to reporter tg338 mice. Transmission with brain or spleen extracts are indicated with black and red lines, respectively. The number of affected/inoculated mice (mice with TSE and positive for brain PrPres by immunoblot) and the mean survival times in days ± SEM are indicated for each inoculated group. Segmented, doubled circles are used to indicate the proportion of mice with 19K PrPres signature (blue) or 21K PrPres signature (grey) in the brain (inside of the circle, black lines) and the spleen (outside of the circle, red lines). (TIF) [file ppat.1008283.s003.tif]
