# Supplementary material for: Host prion protein expression levels impact prion tropism for the spleen
Source: PLoS Pathog. 2020 Jul 23;16(7):e1008283. doi: 10.1371/journal.ppat.1008283 (PMC7402522; doi:10.1371/journal.ppat.1008283)
Supplement: S1 Table — (PDF) [file ppat.1008283.s004.pdf]

Supplementary Table 1. TSE sources transmitted to tg338 mice.

TSE Sources	Reference	Source[1]	Genotype[2]	Reference
PG127	PG127	VLA (UK)	AHQ/VRQ	[1]
LAN group	LAN404	INRA (France)	VRQ/VRQ	[1, 2]
	ARQ16	CVI (Netherlands)	ARQ/ARQ	[3]
	N114	INIA (Spain)		
	N799	INIA (Spain)		
	sc41	CVRL (Eire)		
	sc46	CVRL (Eire)		
	sc47	CVRL (Eire)		
	sc55	CVRL (Eire)		
	99-378	ANSES (France)		
	99-454	ANSES (France)		
	O71	ANSES (France)	VRQ/ARQ	
	O76	ANSES (France)		
	O78	ANSES (France)	VRQ/ARH	
	O171	ANSES (France)		[4]
	O104	ANSES (France)	VRQ/VRQ	[4]
	TR015883	ANSES (France)	ARQ/ARQ	
	TR270589	ANSES (France)	ARQ/ARQ	
	PS165	INRA (France)		
	PS1	INRA (France)		
	PS310	INRA (France)	ARQ/ARQ	[5]
	PS13	INRA (France)	ARQ/ARQ	
	PS16	INRA (France)	ARH/ARH	
	PS17	INRA (France)	ARQ/VRQ	
	PS18	INRA (France)		
	PS83	INRA (France)	ARR/ARR	[6]
	PS137	INRA (France)		
CH1641-like	V418	VLA (Cyprus)		
	06-287	ANSES (France)	ARQ/ARQ	[7, 8]
	06-412	ANSES (France)	ARQ/ARQ	[7, 8]
	06-017	ANSES (France)	ARQ/ARQ	[7, 8]
	05-825	ANSES (France)	ARQ/ARQ	[7, 8]
	UK-2	VLA (UK)	ARQ/ARQ	[9]
	O100	ANSES (France)	VRQ/VRQ	[8]
	O638	ANSES (France)	ARQ/ARQ	
	TR316211	ANSES (France)	ARQ/ARQ	[4]
	PS48	INRA (France)	VRQ/VRQ	[5]
	PS227	INRA (France)	VRQ/VRQ	[10]
	PS303	INRA (France)	VRQ/VRQ	[10]

[1]Supplying laboratory (original country TSE source).

[2]Sheep PrP genotype (when available) for both PrP alleles at codon 136 (A = alanine or V = valine), 154 (R = arginine or H = histidine) and 171 (Q = glutamine, R = arginine, or H = histidine)

References

1. Vilotte JL, Soulier S, Essalmani R, Stinnakre MG, Vaiman D, Lepourry L, et al. Markedly increased susceptibility to natural sheep scrapie of transgenic mice expressing ovine prp. J Virol. 2001;75(13):5977-84. doi: 10.1128/JVI.75.13.5977-5984.2001. PubMed PMID: 11390599; PubMed Central PMCID: PMCPMC114313.
2. Le Dur A, Lai TL, Stinnakre MG, Laisne A, Chenais N, Rakotobe S, et al. Divergent prion strain evolution driven by PrPC expression level in transgenic mice. Nat Commun. 2017;8:14170. doi: 10.1038/ncomms14170. PubMed PMID: 28112164; PubMed Central PMCID: PMCPMC5264111.
3. Langeveld JP, Jacobs JG, Erkens JH, Baron T, Andreoletti O, Yokoyama T, et al. Sheep prions with molecular properties intermediate between classical scrapie, BSE and CH1641-scrapie. Prion. 2014;8(4):296-305. Epub 2014/12/20. doi: 10.4161/19336896.2014.983396. PubMed PMID: 25522672; PubMed Central PMCID: PMCPMC4601226.
4. Baron T, Bencsik A, Morignat E. Prions of ruminants show distinct splenotropisms in an ovine transgenic mouse model. PLoS One. 2010;5(4):e10310. doi: 10.1371/journal.pone.0010310. PubMed PMID: 20436680; PubMed Central PMCID: PMCPMC2859945.
5. Cassard H, Torres JM, Lacroux C, Douet JY, Benestad SL, Lantier F, et al. Evidence for zoonotic potential of ovine scrapie prions. Nat Commun. 2014;5:5821. doi: 10.1038/ncomms6821. PubMed PMID: 25510416.
6. Groschup MH, Lacroux C, Buschmann A, Luhken G, Mathey J, Eiden M, et al. Classic scrapie in sheep with the ARR/ARR prion genotype in Germany and France. Emerg Infect Dis. 2007;13(8):1201-7. Epub 2007/10/24. doi: 10.3201/eid1308.070077. PubMed PMID: 17953092; PubMed Central PMCID: PMCPMC2828083.
7. Baron T, Bencsik A, Vulin J, Biacabe AG, Morignat E, Verchere J, et al. A C-terminal protease-resistant prion fragment distinguishes ovine "CH1641-like" scrapie from bovine classical and L-Type BSE in ovine transgenic mice. PLoS Pathog. 2008;4(8):e1000137. doi: 10.1371/journal.ppat.1000137. PubMed PMID: 18769714; PubMed Central PMCID: PMCPMC2516186.
8. Baron T, Biacabe AG. Molecular behaviors of "CH1641-like" sheep scrapie isolates in ovine transgenic mice (TgOvPrP4). J Virol. 2007;81(13):7230-7. doi: 10.1128/JVI.02475-06. PubMed PMID: 17442721; PubMed Central PMCID: PMCPMC1933328.
9. Beck KE, Sallis RE, Lockey R, Vickery CM, Beringue V, Laude H, et al. Use of murine bioassay to resolve ovine transmissible spongiform encephalopathy cases showing a bovine spongiform encephalopathy molecular profile. Brain Pathol. 2012;22(3):265-79. Epub 2011/09/17. doi: 10.1111/j.1750-3639.2011.00526.x. PubMed PMID: 21919992; PubMed Central PMCID: PMCPMC3505794.
10. Tang Y, Gielbert A, Jacobs JG, Baron T, Andreoletti O, Yokoyama T, et al. All major prion types recognised by a multiplex immunofluorometric assay for disease screening and confirmation in sheep. J Immunol Methods. 2012;380(1-2):30-9. Epub 2012/04/14. doi: 10.1016/j.jim.2012.03.004. PubMed PMID: 22498749.
