# Supplementary material for: Host prion protein expression levels impact prion tropism for the spleen
Source: PLoS Pathog. 2020 Jul 23;16(7):e1008283. doi: 10.1371/journal.ppat.1008283 (PMC7402522; doi:10.1371/journal.ppat.1008283)
Supplement: S2 Table — (PDF) [file ppat.1008283.s005.pdf]

Supplementary Table 2. PrP^{res} signature in the brain and spleen after intraperitoneal inoculation of LAN, CH1641-like isolates and LA19K prions to tg338 mice

Isolate	Survival time		Ratio	Spleen PrP^{res} type		Brain PrP^{res} type[b] (n/n$_0$)[d]
	IC route (n/n$_0$)[a]	IP route (n/n$_0$)[a]	IP/IC	Detection from→up to	PrP^{res} type[b] (n/n$_0$)[c]	
PG127	74 ± 1 (4/4)	97 ± 3 (8/8)	x 1.3	30 d → end stage/life	21K (15/15)	21K (6/6)
LAN404	*199 ± 10 (10/10)*	553 ± 26 (7/7)	x 2.8	50 d[e] → end stage/life	21K (18/18)	21K (4/7); 19K (2/7)
ARQ16	133 ± 8 (6/6)	566 ± 51 (12/12)	x 4.2	100 d[e] → end stage/life	21K (20/20)	21K (2/10); 19K (4/10)
99-378	195 ± 21 (6/6)	506 ± 40 (10/10)	x 2.6	100 d[e] → end stage/life	21K (15/15)	21K (6/8)
99-454	136 ± 2 (6/6)	532 ± 27 (12/12)	x 3.9	100 d[e] → end stage/life	21K (20/20)	21K (9/10); 19K (1/10)
O100	177 ± 9 (7/7)[f]	553 ± 25 (7/7)	x3.1	200 d[e] → end stage/life	21K (12/12)	21K (5/7)
48	141 ± 1 (6/6)	556 ± 29 (8/8)	x3.9	100 d[e] → end stage/life	21K (12/12)	21K (3/7); 19K (1/7)
LA19K (4p)	*129 ± 3 (6/6)*	392 ± 56 (8/8)	x3.0	20 d → end stage/life	21K (17/17)	21K (5/7); 19K (1/7)
LA19K (6p)	131 ± 1 (15/15)	462 ± 33 (27/27)	x3.5	50 d[e] → end stage/life	21K (40/40)	21K (12/27); 19K (16/27)[g]
Cl-LA19K[h]	134 ± 2 (7/7)	354 ± 42 (6/8)	x2.6		neg (11/11)	19K (6/8)

d: days; Neg: negative; TS: terminal stage of disease
[a]Number of mice with disease or PrP^{res}-positive in brain or spleen/number of mice inoculated
[b]As referred to the size of unglycosylated PK-resistant PrP^{Sc} in immunoblots
[c]Number of mice with the PrP^{res} type found/number of mice tested (kinetic analysis)
[d]Number of mice with the PrP^{res} type found/number of mice tested (end stage/life)
[e]Not tested before the indicated time
[f]1% infection
[g]One mixed profile observed
[h]Use as inoculum LA19K cloned twice by limiting dilution
