# Supplementary material for: Host prion protein expression levels impact prion tropism for the spleen
Source: PLoS Pathog. 2020 Jul 23;16(7):e1008283. doi: 10.1371/journal.ppat.1008283 (PMC7402522; doi:10.1371/journal.ppat.1008283)
Supplement: Attachment — Submitted filename: Rebuttal.docx [file ppat.1008283.s006.docx]

Please find below our point by point response to the referee’s comments.

Description of the changes made in the manuscript:

We profoundly restructured the whole manuscript to improve its clarity. We changed the content and numbers of paragraphs in the results section to streamline the findings and make the understanding of our experiments easier. At the beginning of each paragraph, when necessary, the rationale of the experiments is better explicated. The main conclusions have also been reworked. For example, we separated the transmission data resulting from primary and serial passaging of LAN prions (for IC and IP inoculations). We made a single paragraph regarding the kinetics of PrPres accumulation in tg338 mouse brain and spleen. The figures have been modified accordingly. There are now 7 figures. We improved their clarity. Some data have been moved as supplementary information to simplify the results. We finally modified the discussion to further strengthen our message and highlight the degree of advance over our previous study published in Nature Communications [1]. We provide as supplementary manuscript the revised manuscript with tracked changes. We do hope that in its present form the revised version will satisfy the issues raised in the initial manuscript.

**Part I - Summary**

Reviewer #1: The authors previously shown that PrPC expression level in transgenic mice can influence the emergence of prion strains. The authors build upon these findings and provide evidence suggesting that differences in PrPC expression between the CNS and LRS also can influence strain tropism and emergence. The authors include an impressive body of transmission data and accompanying biochemical analysis to support this finding. Similar findings were observed using the scrapie and CDJ isolates strengthens the findings in the transgenic mouse system. Overall the conclusions are supported by the data. The discussion is thoughtful and the suggestion that the inclusion of PrPSc properties from spleen when stain typing is significant. Suggestions to improve the manuscript would be to include quantification and statistical analysis of PrPSc levels where conclusions are based on differences in abundance in figure 1D and Figure 6. The authors should double check the formatting of the references, some errors were noted.

Thank you for these comments. As proposed, we now include quantification of PrPSc levels in Figure 1D (now figure 3) and figure 6 (now figure 7). We did not include statistics in Figure 7 as no differences are observed. We also corrected the errors in the references.

Reviewer #2: The goal of this study by Beringue et al. was to uncover the underlying mechanism of strain tropism and selective replication of substrains in spleen and brain tissue, respectively. They propose that the PrPC expression level, which is about 20fold lower in the spleen than in the brain, determines replication, and demonstrate that in the spleen of transgenic mice overexpressing ovine PrP (tg338) LA21K prions are found, whereas in the brains of those mice mostly LA19K prions replicate. The same group has published earlier that strain selection in the brain is linked to PrPC expression levels, with LA21K replicating in the brains of low/physiological-level PrPC-expressing mice, while propagation of LA19K required high expression levels of PrPC (LeDur et al., Nature Communications 2017). The results of the current study re-iterate the importance of PrPC expression levels for the replication of specific substrains. However, it does not address other potential factors that might drive tissue tropism (eg. tissue-specific co-factors or PrPC modifications, conformational differences of PrPSc).

We first show that the two substrains components we previously identified in LAN sheep scrapie isolates segregate between brain and spleen in a single infected mouse, which was unexpected. We further show that CH1641-like isolates are composed of these 2 substrains and behave similarly. Such spleen/brain segregation offered a unique paradigm to address the mechanisms of prion tropism for the lymphoid tissue.

Using these isolates and CJD-derived co-propagating substrains, we then found a strong correlation between capacity to replicate in the spleen and ease to propagate in mice expressing low levels of PrP^C^. Oppositely, strains with high PrPC needs to replicate in the brain may not be able to replicate in the spleen.

This correlation leads us to propose that the low-PrP^C^ expression levels in the spleen may govern prion tropism for this tissue. There would thus be no need for other co-factors (from the host or the strain). We discuss the possible contribution of potential host co-factors or PrP^Sc^ conformations in the discussion section. However, none of the previously reported hypothesis would fully support our data.

Beyond the new core message that host PrP^C^ levels can drive prion tropism for the lymphoid tissue, we deliver important message regarding prion evolution. We provide evidence that PrP^C^ levels variations may be a driver of within-host prion diversification/evolution; the selection pressure imposed by such variations can be as strong as that observed during cross-species transmission events with selection of a unique substrain. Overall our study raises the question of the biological importance of within-host prion diversification and highlights the possible contribution of co-propagating substrains on prion disease kinetics and pathological characteristics in the infected hosts. This is an important issue given that many naturally infected species, including humans, contains mixture of prion substrains. We do hope that these elements will convince you that our study is far from being a re-iteration of our Nature Communications paper.

Reviewer #3: In this manuscript the authors attempt to discriminate and characterize two ovine strains through subsequent passages in transgenic (Tg) mice overexpressing sheep prion protein (PrP). The biochemical and neuropathological characterization and passaging strategies have identified a putative correlation with the expression levels of PrP in either brain or spleen.

**Part II – Major Issues: Key Experiments Required for Acceptance**

Reviewer #1: Suggestions to improve the manuscript would be to include quantification and statistical analysis of PrPSc levels where conclusions are based on differences in abundance in figure 1D and Figure 6.

Quantifications are now included in the revised version as Figure 3 and Figure 7.

Reviewer #2: The manuscript overall was difficult to follow and it was not well justified why so many different isolates were used, or even different passages of LA19K (Fig. 3C).

We use many isolates for primary transmission to provide scientifically solid observations and a consistent picture of the PrP^res^ profiles in brain and spleen tissue on primary or subsequent passage.

As example, the reviewer may refer to the transmission of the ARQ16 isolate by IP route. All the brain tested 19K-PrP^res^ whereas with the other LAN isolates, the proportion of brains tested 19K-PrP^res^ was low. Using ARQ16 alone would have conveyed incomplete or even false information. Transmitting a panel of isolates allowed a broader qualitative and quantitative analysis of the PrP^res^ patterns in brain and spleen and overall strengthened the validity of our data.

We applied the same strategy when inoculating LAN-passaged prions by IP route given the variations observed on primary passage regarding the disease tempo, the clinical manifestation of the disease and the dominant strain type replicating in the brain.

These justifications are now included in the revised version.

PMCA would have been helpful to compare replication rates in the different tissues.

This is a sound remark. However, LA19K prions are refractory to amplification by PMCA using tg338 brain as substrate. We did not achieve sufficiently high prion amplification rate using spleen PrP^C^ as substrate to perform comparative studies with brain substrate. When necessary, we performed bioassays (e.g. titration of spleens infected with cloned LA19K prions).

Some statements are not supported by the results that are shown:
p. 13, lanes 4, 5 and Figure 1D: it is difficult to verify that accumulation of 21K-PrPres plateaued from 60 days post infection in the spleen. There is a clear increase to day 100, and at the terminal stage twice the amount of protein was loaded; the same is true for the panels showing brain PrPres, similar amounts of proteins should be loaded to allow comparison of propagation dynamics.

We now provide a precise quantification of PrP^res^ levels in brain and spleens (Figure 3). As correctly noted by the reviewer, there is no plateau of LA21K PrP^res^ accumulation in the spleen. This is quite unusual as with other strains or models there is a cessation of PrP^res^ accumulation in the spleen with time.

Figure 2B: the bands detected in brain and spleen from spleen-inoculated mice (lanes 3 and 4) is described as 21K-PrPres, however, it migrates faster than the 21K-PrPres in the brains of tg143+/-. Confirmation with 12B2 antibody would be helpful.

Thank for this comment. Spleen PrP^res^ is known to migrate slightly faster than brain PrP^res^. Typically, there is a 0.5-1 kDa difference [2-9]), irrespective of the host and TSE agent (PrP^C^ migrates slightly faster than brain PrP^C^ [2]). A sentence has been added in Figure 1 legend. However, to avoid any ambiguity we now provide blots with 12B2 antibody which confirms the 21K pattern.

Figure 2C: the background signal in the lower panel is much higher, making it difficult to compare the appearance of PrPSc deposits.

The brain sections have been reacquired to provide similar background signals in the top and bottom panels.

Figure 4B: the PrPres band in lane 5 (i.c./Sp) is supposed to be 21K but appears to migrate faster than the 21kDa-pattern in lane 1; it is stated on p. 17/lane 14 that i.p. inoculation resulted in 21K-PrPres in brains and spleens, but the migration pattern in lanes 2 and 3 differs; re-probing with 12B2 might clarify the banding pattern. Further, the legend and figure annotation for lane 1 needs to be clarified.

See comments above. The blots have been reprobed with 12B2 and confirm a 21K pattern.

Figure 6: The heading of the legend does not fit with the results shown. The statement that PK resistance and degradation rate of LA19K and 127S (what is the source of 127S?) are not different is not supported by the data. LA19K is detectable even with 10 mg/ml of PK and might be more resistant even to macrophage degradation, with a faint signal after 10 days. Quantification and statistical analysis is needed to draw a conclusion.

Thank you for pointing that. The heading has been modified. The origin of 127S is now mentioned (serial passage and cloning of PG127 sheep isolate in tg338 mice).

We now provide quantification of the data. We also rephrased the discussion section regarding these experiments to make it clearer that LA19K prions are as resistant as 127S splenotropic prions to degradation.

Reviewer #3: I had a very difficult time in reading this account. I think the data are solid and support the conclusion drawn in the discussion but it is a daunting task to actually understand what the authors sometime want to express in their writing.
I would like to suggest to completely re-write the manuscript and streamline the findings in an more methodical and systematic way.
This amount of experimental data deserve a clearer account of the findings and conclusions.

 We have profoundly restructured the manuscript and figures and do hope that the findings and message are clearer.

**Part III – Minor Issues: Editorial and Data Presentation Modifications**


Reviewer #1: The authors should double check the formatting of the references, some errors were noted.

Reviewer #2: Figure 6: The heading of the legend does not fit with the results shown.
p. 17, lane 14: results shown in Figure 4A - B are described, the authors refer to Figure 3A-B though.

Reviewer #3: Figures are difficult to follow. Too much information in a very short space. I would like to suggest to revise the presentation of results.

This has been corrected, thank you.

References

1.	Le Dur A, Lai TL, Stinnakre MG, Laisne A, Chenais N, Rakotobe S, et al. Divergent prion strain evolution driven by PrPC expression level in transgenic mice. Nat Commun. 2017;8:14170. doi: 10.1038/ncomms14170. PubMed PMID: 28112164; PubMed Central PMCID: PMCPMC5264111.

2.	Beringue V, Herzog L, Jaumain E, Reine F, Sibille P, Le Dur A, et al. Facilitated cross-species transmission of prions in extraneural tissue. Science. 2012;335(6067):472-5. Epub 2012/01/28. doi: 10.1126/science.1215659

335/6067/472 [pii]. PubMed PMID: 22282814.

3.	Chapuis J, Moudjou M, Reine F, Herzog L, Jaumain E, Chapuis C, et al. Emergence of two prion subtypes in ovine PrP transgenic mice infected with human MM2-cortical Creutzfeldt-Jakob disease prions. Acta neuropathologica communications. 2016;4(1):2-15. doi: 10.1186/s40478-016-0284-9.

4.	Langevin C, Andreoletti O, Le Dur A, Laude H, Beringue V. Marked influence of the route of infection on prion strain apparent phenotype in a scrapie transgenic mouse model. Neurobiol Dis. 2011;41(1):219-25. Epub 2010/09/30. doi: S0969-9961(10)00311-6 [pii]

10.1016/j.nbd.2010.09.010. PubMed PMID: 20875860.

5.	Dron M, Moudjou M, Chapuis J, Salamat MK, Bernard J, Cronier S, et al. Endogenous proteolytic cleavage of disease-associated prion protein to produce C2 fragments is strongly cell- and tissue-dependent. J Biol Chem. 2010;285(14):10252-64. doi: 10.1074/jbc.M109.083857. PubMed PMID: 20154089; PubMed Central PMCID: PMCPMC2856230.

6.	Baron T, Bencsik A, Morignat E. Prions of ruminants show distinct splenotropisms in an ovine transgenic mouse model. PLoS One. 2010;5(4):e10310. doi: 10.1371/journal.pone.0010310. PubMed PMID: 20436680; PubMed Central PMCID: PMCPMC2859945.

7.	Beringue V, Adjou KT, Lamoury F, Maignien T, Deslys JP, Race R, et al. Opposite effects of dextran sulfate 500, the polyene antibiotic MS-8209, and Congo red on accumulation of the protease-resistant isoform of PrP in the spleens of mice inoculated intraperitoneally with the scrapie agent. J Virol. 2000;74(12):5432-40. PubMed PMID: 10823847; PubMed Central PMCID: PMCPMC112027.

8.	Davenport KA, Christiansen JR, Bian J, Young M, Gallegos J, Kim S, et al. Comparative analysis of prions in nervous and lymphoid tissues of chronic wasting disease-infected cervids. J Gen Virol. 2018. Epub 2018/03/28. doi: 10.1099/jgv.0.001053. PubMed PMID: 29580373.

9.	Wadsworth JD, Joiner S, Hill AF, Campbell TA, Desbruslais M, Luthert PJ, et al. Tissue distribution of protease resistant prion protein in variant Creutzfeldt-Jakob disease using a highly sensitive immunoblotting assay. Lancet. 2001;358(9277):171-80. Epub 2001/07/31. doi: 10.1016/s0140-6736(01)05403-4. PubMed PMID: 11476832.
